# Evaluation of the Combination of Artificial Intelligence and Radiologist Assessments to Interpret Malignant Architectural Distortion on Mammography

**DOI:** 10.3389/fonc.2022.880150

**Published:** 2022-04-20

**Authors:** Yun Wan, Yunfei Tong, Yuanyuan Liu, Yan Huang, Guoyan Yao, Daniel Q. Chen, Bo Liu

**Affiliations:** ^1^ Department of Radiology, The Second Affiliated Hospital of Guangzhou University of Chinese Medicine, Guangzhou, China; ^2^ AI Research Lab, Boston Meditech Group, Burlington, MA, United States; ^3^ AI Research Lab, Shanghai Yanghe Huajian Artificial Intelligence Technology Co., Ltd, Shanghai, China

**Keywords:** artificial intelligence, architectural distortion, mammography, breast cancer, malignant

## Abstract

**Purpose:**

To compare the mammographic malignant architectural distortion (AD) detection performance of radiologists who read mammographic examinations unaided versus those who read these examinations with the support of artificial intelligence (AI) systems.

**Material and Methods:**

This retrospective case-control study was based on a double-reading of clinical mammograms between January 2011 and December 2016 at a large tertiary academic medical center. The study included 177 malignant and 90 benign architectural distortion (AD) patients. The model was built based on the ResNeXt-50 network. Algorithms used deep learning convolutional neural networks, feature classifiers, image analysis algorithms to depict AD and output a score that translated to malignant. The accuracy for malignant AD detection was evaluated using area under the curve (AUC).

**Results:**

The overall AUC was 0.733 (95% CI, 0.673-0.792) for Reader First-1, 0.652 (95% CI, 0.586-0.717) for Reader First-2, and 0.655 (95% CI, 0.590-0.719) for Reader First-3. and the overall AUCs for Reader Second-1, 2, 3 were 0.875 (95% CI, 0.830-0.919), 0.882 (95% CI, 0.839-0.926), 0.884 (95% CI, 0.841-0.927),respectively. The AUCs for all the reader-second radiologists were significantly higher than those for all the reader-first radiologists (Reader First-1 vs. Reader Second-1, P= 0.004). The overall AUC was 0.792 (95% CI, 0.660-0.925) for AI algorithms. The combination assessment of AI algorithms and Reader First-1 achieved an AUC of 0.880 (95% CI, 0.793-0.968), increased than the Reader First-1 alone and AI algorithms alone. AI algorithms alone achieved a specificity of 61.1% and a sensitivity of 80.6%. The specificity for Reader First-1 was 55.5%, and the sensitivity was 86.1%. The results of the combined assessment of AI and Reader First-1 showed a specificity of 72.7% and sensitivity of 91.7%. The performance showed significant improvements compared with AI alone (p<0.001) as well as the reader first-1 alone (p=0.006).

**Conclusion:**

While the single AI algorithm did not outperform radiologists, an ensemble of AI algorithms combined with junior radiologist assessments were found to improve the overall accuracy. This study underscores the potential of using machine learning methods to enhance mammography interpretation, especially in remote areas and primary hospitals.

## Introduction

Breast cancer has become the most commonly diagnosed cancer in the world, overtaking lung cancer. There were 2.26 million new breast cancer cases in 2020, and 68.5 hundred thousand patients died (World Health Organization International Agency for Research on Cancer, IARC) (1). Breast cancer has become the most common malignant tumors among Chinese women, accounting for approximately 15% of all female cancers, and its overall mortality rate has increased in recent years (2). Breast cancer screening with mammography is considered effective at reducing breast cancer-related mortality (3–6). Currently, mammograms are subjectively interpreted by radiologists and rely heavily on their qualitative visual experience to identify relevant traits (7); thus, the benefit of mammograms is dependent on subjective human interpretation to maximally extract all diagnostic information from the acquired images (8). However, mammography screening is imperfect, as the identification of subtle lesions is challenging; as a result, 12.5% of malignancies are missed in clinical practice (9, 10). In contrast to masses and calcifications, architectural distortion (AD) is the most difficult type of tumor to detect and the most commonly missed abnormality due to its inherent subtlety and varying attributes.

Architectural distortion on mammography, defined as distortion of the breast parenchymal architecture without a definable mass, can be due to malignant lesions, such as invasive cancer or ductal carcinoma *in situ* (DCIS), or to benign lesions, such as a radial scar or complex sclerosing lesion (11). Architectural distortion (AD) has been described by the American College of Radiology in Breast Imaging Reporting and Data System (BI-RADS) as follows: “For mammography, this includes thin straight lines or spiculations radiating from a point, and focal retraction, distortion, or straightening at the anterior or posterior edge of the parenchyma” (12). AD can be associated with calcifications and asymmetries; therefore, cases with associated masses were excluded. Furthermore, the orientation of linear structures within AD lesions, such as ligaments, ducts, and blood vessels, may mimic normal anatomical variations in breast tissue texture, making perception particularly difficult. Visually, both benign and malignant AD appear to be more or less the same. Many times, readers report that they perceive an abnormality, but they are often unable to make more accurate decisions to differentiate between benign and malignant tissues, especially for radiologists with little experience in mammography.

Artificial intelligence (AI), powered by recent advances in machine learning, may make computer-aided diagnosis (CAD) for mammography more valuable in clinical practice (7). The most promising of these advances is deep learning, a family of machine learning methods focusing on developing convolutional neural networks (8, 13). Radiologists have been able to improve their cancer detection and risk prediction by mammography when using an AI system for support (14, 15). Several articles have reported the detection of AD in radiomics analyses (16–21). However, few studies have used deep learning or focused on developing multilayered neural networks.

The purpose of our study was to assess whether AI algorithms can overcome the limitations of human mammography interpretation, match radiologists’ interpretations of AD on mammography performance and improve the interpretive accuracy.

## Materials and Methods

This retrospective study was approved by our institutional review board, and written informed consent was waived. Women were included from one institution (The Second Affiliated Hospital of Guangzhou University of Chinese Medicine).

### Study Population

We collected consecutive digital clinical mammograms (Hologic, Bedford, Mass) between January 2011 and December 2016 at a large tertiary academic medical center. For each patient, we obtained outcomes through linkage to tumor registries at four hospitals within our health care system, supplemented with pathologic findings from our mammography information system electronic medical records (Y.L. Z Version 8.0.143; Md).

### Case Collection

We indicated women with architectural distortion on mammography. A total of 177 subjects had pathologically confirmed breast cancer. Ninety benign results were pathologically confirmed as benign, or no cancer was diagnosed followed for 2 years. Exclusion criteria included a history of breast cancer or prior surgery.

### Population Characteristics

The population characteristics and the digital mammographic examinations included for the observer study are shown in **Table 1**. All digital mammographic examinations were bilateral and contained two views (craniocaudal and mediolateral oblique). Cancer cases were verified by means of histopathologic evaluation. A total of 177 patients had malignant tumors, including 124 cases of invasive ductal carcinoma, 38 cases of ductal carcinoma in situ, and 15 cases of invasive lobular carcinoma.

**Table 1:** Characteristics of the population and digital mammographic examinations selected for the study.

Variable	177 subjects with malignant architecture distortion	90 subjects with benign results
Patient age (y)		
Mean	49.51±9.12	48.18±7.65
Median	49	47
Range	27-79	34-84
Interquartile range	43-56	43-52
BI-RADS breast density		
a	0	2
b	11	8
c	162	68
d	4	11

### Observation Evaluation

A fully crossed, multi-reader, multi-case evaluation with two sessions (separated by at least 4 weeks) was performed to test both reading conditions. There were 3 different first-reader radiologists and 3 different second-reader radiologists. The first readers were general radiologists, and the second readers were breast radiologists. The median experience with mammography diagnosis of the first readers was three years (range, 2-4years), and the approximate mean number of mammograms read per year during the past 2 years was 200 (range,150-300). The median experience with mammography diagnosis of the second readers was 10 years (range, 8-12 years), and the approximate mean number of mammograms read per year during the past 2 years was 4800 (range, 4500-5000). In addition, when performing the assessments, the second readers could access the assessment already performed by the first reader.

Radiologists were blinded to any information about the patient, including previous radiology and histopathology reports. Before the first session, each radiologist was individually trained in a session with 30 examinations not included in the final evaluation. The training was intended to familiarize radiologists with the evaluation workstation, the evaluation criteria, and the AI support system (e.g., to understand how to use all its functionalities).

For each examination, the radiologists provided a forced Breast Imaging Reporting and Data System (BI-RADS) score (range, 1–5) and assigned a probability of malignancy (POM) between 1 and 100 (with 100 indicating highly suspicious for malignancy). During training, radiologists were instructed to use the full extent of the POM scale with anchor points as a guide. For instance, a BI-RADS category of 2 was recommended at a POM of 20, a BI-RADS category of 3 was recommended at a POM of 30, and the transition from a BI-RADS category of 4a to 4c was recommended at a POM of 50, 60, and 70. A BI-RADS category of 5 was recommended at a POM of 80.

### AI Support System

Using ITK-Snap software, two breast radiologists with rich experience (Y.Y.L with 15 years of experience and Y.W. with 18 years of experience) independently manually delineated the AD on mammography. To avoid introducing more noise and non-AD areas, doctors are required to mark the core area of AD as much as possible. Then, we calculated the intersection and association ratio (IOU) of the two regions of interest to evaluate the degree of overlap. If the IOU ≤ 0.5, the contour area should be re-evaluated, and the contour target area with high consistency was ultimately input into the model. The model was built based on the ResNext-50 network. We rotated each image by -10°, -5°, 5° and 10° and then transformed the images by adding noise to improve the model robustness (22, 23). To enhance image contrast, we used top and bottom hat transform and gamma transform to make the bright areas of the image brighter and the dark areas darker.

**Figure 1:**
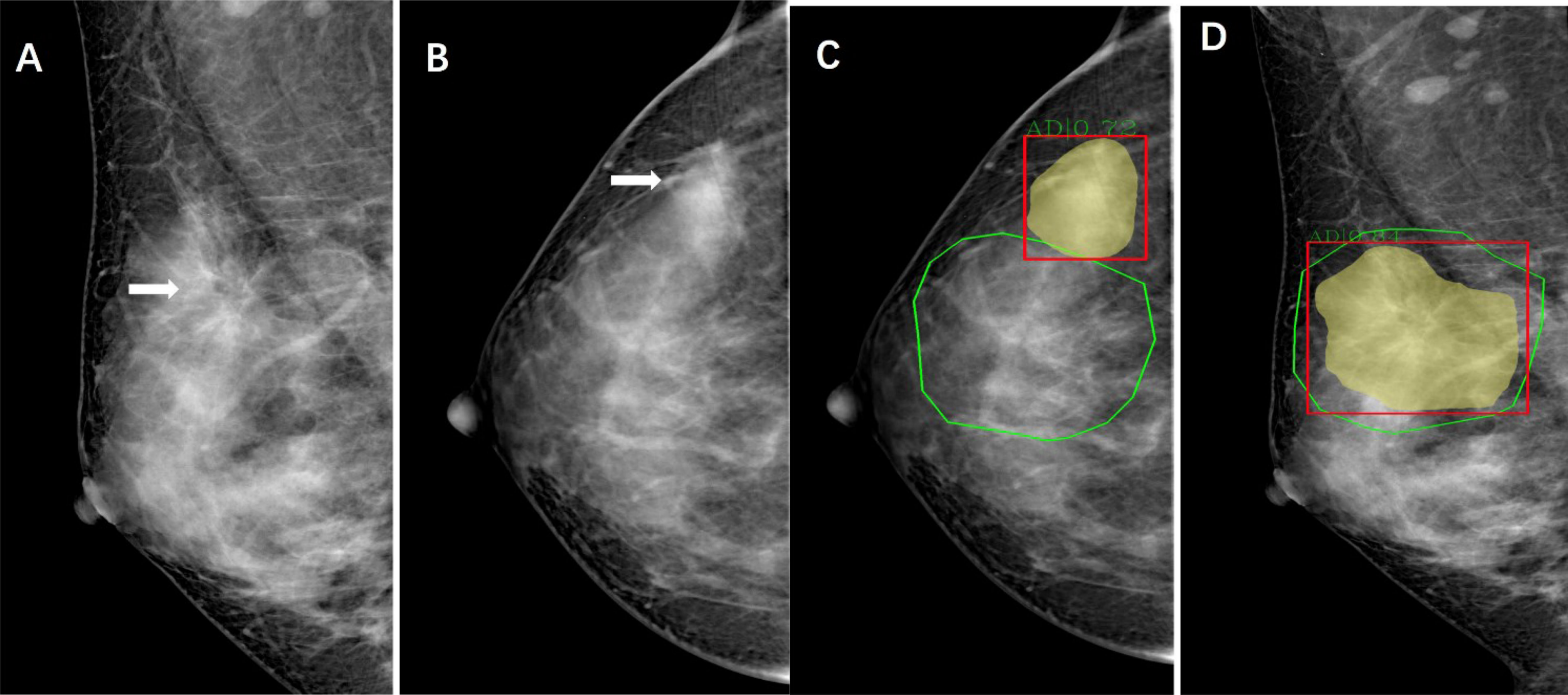
Images of a 44-year-old woman with architectural distortion who presented for clinical mammography. **(A)** Right mediolateral oblique mammogram shows malignant architectural distortion (arrow) in the upper outer quadrant. **(B)**, Right craniocaudal mammogram shows an AD (arrow) with increased gland density. **(C, D)** Green outlined areas were manually delineated for architectural distortion on mammography by radiologists using ITK-Snap software. Yellow and read outlined areas and scores are shown as observed in the viewer of the AI system.

The system uses deep learning convolutional neural networks and feature classifiers and image analysis algorithms to depict AD in two different modules. Each individual algorithm outputs a confidence level (a number between 0 and 1) indicative of the likelihood estimated by the algorithm representing the level of suspicion that cancer is present (with 1 indicating the highest suspicion) (**Figure 1**). Finally, proprietary algorithms are used to combine the scores of the detected regions in craniocaudal and/or mediolateral oblique right and/or left breast images into an examination-based score ranging from 1 to 10 (with 10 indicating the highest likelihood that cancer is present on the mammogram).

### Model Construction

All the patients in the two groups (malignant, benign) were randomly divided into a training set, verification set and test set at a ratio of 6:2:2. The training set included 105 cancer and 54 benign cases, and the verification set and test set included 36 cancer and 18 benign cases randomly. ResNeXt was selected as the basic network of Mask R-CNN, and the Feature Pyramid Network (FPN) was connected behind the basic network (24). For our Mask RCNN, the input size is 1280 x 780. The initial learning rate is set 0.02 and the loss functions for classification and mask are both cross entropy function. For bound box regression, we use L1 loss. For the region proposal network, we use multiple scales for anchors, which are set 8, 16, 32 with ratios 0.5, 1.0 and 2.0 respectively. Such settings can cover the whole image. When we select anchors, the regions that have more than 0.7 overlaps are selected as positive samples while those with less than 0.3 overlaps are selected as negative samples. We set random sample number be 256 to control the number of proposals. We iteratively train our model and periodically evaluate the model on our validation set. The model that performs best on our validation set was selected as the final model.

By integrating the features, the feature perception ability of the network at different scales was obtained. Receiver operating characteristic (ROC) curves were drawn, and the model performance was evaluated by the area under the ROC curve (AUC), accuracy, sensitivity, and specificity.

We determined performance levels for AI algorithms and for all radiologists’ assessments (Reader First, Reader Second, and Consensus) in the patients from the test set for the following diagnostic metrics: sensitivity, specificity, accuracy, positive predictive value (PPV), and negative predictive value (NPV).

We also investigated whether an association existed between the number of abnormal interpretations and the number of cases positive for cancer detected by the AI algorithms alone and combined with the assessment of the Reader First and Reader Second as well as the Reader First and Reader Second consensus. When performing the consensus assessment, the readers can access the assessment already performed by the AI and make a final diagnosis. We also examined the sensitivity and specificity for the AI CAD algorithm and radiologist combination.

### Statistical Analysis

The main end points of the study were to compare the area under the receiver operating characteristic (ROC) curve, sensitivity and specificity. The area under the ROC curve (AUC), specificity and sensitivity values were compared between reading conditions by using mixed-model analysis of variance and generalized linear models for multiple repeated measurements.

Statistical analysis was performed with SPSS software (version 24; IBM, Armonk, NY), MedCalc software (version 19.1;Mariakerke,Belgium) and GraphPad Prism 9 (GraphPad Software Inc., San Diego, CA, USA).

## Results

### Reader First Performance

**Table 2** reports the AUC values for malignant AD detection for each First Reader overall and by subgroup. The overall AUC was 0.733 (95% CI, 0.673-0.792) for Reader First-1, 0.652 (95% CI, 0.586-0.717) for Reader First-2, and 0.655 (95% CI, 0.590-0.719) for Reader First-3. The differences between Reader First-1 and each other reader first radiologists (Reader First-2 and Reader First-3) were statistically significant (P =0.014, 0.015, respectively), whereas there was no significant difference between Reader First-2 and Reader First-3 (P = 0.934). In addition, we observed that the AUCs for younger vs. older and for higher vs. lower breast density were significantly lower for all first readers. For Reader First-1, the AUC values were 0.768 for women 55 years or older and 0.723 for women younger than 55 years, and the AUC values were 0.730 for mammograms with a high density percentage and 0.748 for mammograms with a low density percentage.

**Table 2:** Area under the receiver operating characteristic curve for the 3 first readers.

Group (n=)	AUC (95%CI)		
	Reader First-1	Reader First-2	Reader First-3
Overall	0.733 (0.673-0.792)	0.652 (0.586-0.717)	0.655 (0.590-0.719)
By age women, Y			
Younger (<55)	0.723 (0.655-0.791)	0.643 (0.569-0.718)	0.643 (0.569-0.716)
Older (≥55)	0.768 (0.651-0.884)	0.677 (0.534-0.819)	0.675 (0.533-0.818)
By mammographic density			
Low	0.748 (0.544-0.952)	0.685 (0.484-0.887)	0.595 (0.362-0.828)
High	0.730 (0.666-0.794)	0.648 (0.576-0.720)	0.660 (0.591-0.729)

### Reader Second Performance

**Figure 2:**
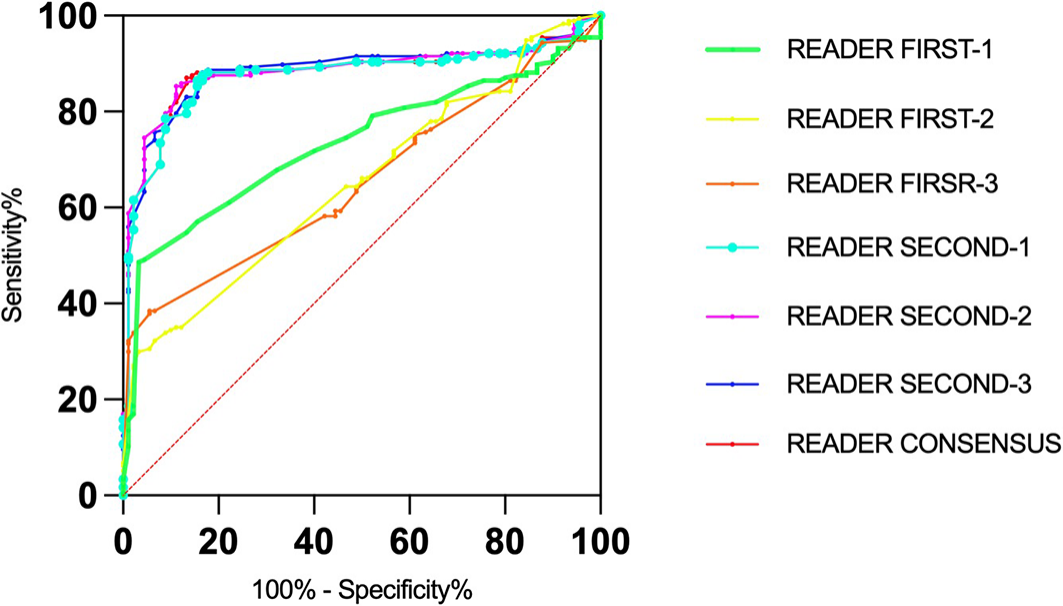
Receiver operating characteristic (roc) curves for the senior and junior readers and consensus.

**Table 3** reports the AUC for malignant AD detection for each reader second radiologist overall and by subgroup. Overall, the AUC was 0.875 (95% CI,0.830-0.919) for Reader Second-1, 0.882 (95% CI,0.839-0.926) for Reader Second-2, and 0.884 (95% CI,0.841-0.927) for Reader Second-3. The AUCs for all the reader second radiologists were significantly higher than those for all the reader-first radiologists (Reader First-1 vs. Reader Second-1, P= 0.004). The differences between Reader Second-1 and each of the other reader second radiologists (Reader Second-2 and Reader Second-3) were not statistically significant (P= 0.237, P= 0.180, respectively), and there was no significant difference between Reader First-2 and Reader First-3 (P = 0.736). Reader First-1 and Reader Second-1 performed a consensus assessment, and the AUC was 0.878 (95% CI, 0.834-0.922) for the consensus discussion. There was no significant difference between Reader Second-1 vs. Consensus discussion (P = 0.113), Reader Second-2 vs. Consensus discussion (P = 0.507), or Reader Second-3 vs. Consensus discussion (P = 0.385). In addition, we observed that the AUCs for younger vs. older and for higher vs. lower breast density were not significantly decreased for all second readers, different to all first readers. The receiver operating characteristic (ROC) curves for individual first readers, second readers and consensus readers unaided by the AI computer system are shown in **Figure 2**.

**Table 3:** Area under the receiver operating characteristic curves for the 3 second and consensus readers.

	AUC (95%CI)			
Group (n=)	Reader Second-1	Reader Second-2	Reader Second-3	Consensus
Overall	0.875 (0.830-0.919)	0.882 (0.839-0.926)	0.884 (0.841-0.927)	0.878 (0.834-0.922)
By age, Y				
Younger (<55)	0.878 (0.828-0.928)	0.888 (0.840-0.935)	0.892 (0.845-0.939)	0.879 (0.830-0.929)
Older (≥55)	0.868 (0.774-0.963)	0.863 (0.782-0.969)	0.863 (0.768-0.959)	0.880 (0.788-0.973)
By mammographic density				
Low	0.863 (0.700-1.000)	0.884 (0.724-1.000)	0.884 (0.724-1.000)	0.868 (0.708-1.000)
High	0.874 (0.827-0.921)	0.884 (0.831-0.924)	0.881 (0.834-0.927)	0.877 (0.830-0.924)

### AI Performance

**Figure 3:**
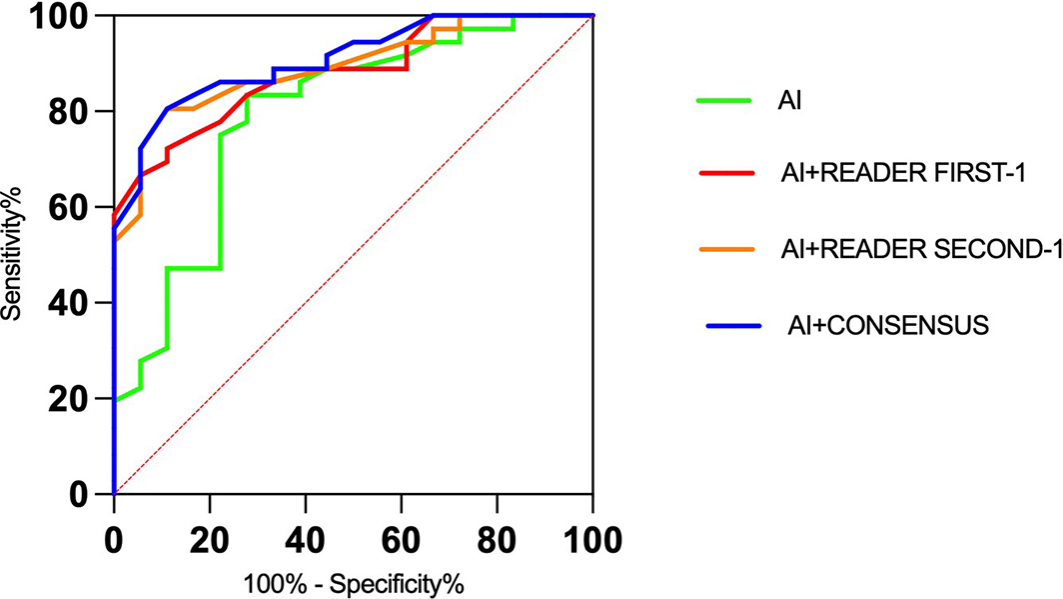
Receiver operating characteristic (ROC) curves for the artificial intelligence algorithms alone and radiologists with the aid of AI algorithms.

**Table 4** presents the AUCs for malignant AD detection for the AI algorithms and for the simulated scenarios in which the binary decisions by the AI algorithms and the readers were combined. Overall, the AUC values were 0.792 (95% CI, 0.660-0.925) for AI algorithms, 0.880 (95% CI, 0.793-0.968) for AI algorithms combined with Reader First-1, and 0.893 (95% CI, 0.809-0.976) for AI algorithms combined with Reader Second-1. The AUC was 0.908 (95% CI, 0.832-0.984) for AI algorithms combined with the consensus discussion of Reader First-1 and Reader Second-1. In addition, we observed that the AUCs for younger vs. older and for higher vs. lower breast density were significantly lower for AI algorithms and AI algorithms combined with radiologist readings. There was no significant difference in the AUC values between AI algorithms vs. Reader First-1 (P=0.493), AI algorithms combined with Reader First-1 vs. AI algorithms combined with Reader Second-1 (P = 0.454), AI algorithms combined with Reader First-1 vs. AI algorithms combined with consensus discussion (P = 0.004). The receiver operating characteristic (ROC) curves for the AI algorithms and Reader First-1, Reader Second-1 and consensus reading mammograms aided with AI computer systems are shown in **Figure 3**.

**Table 4:** Area under the receiver operating characteristic curves for the artificial intelligence algorithms and for algorithms combined with the assessment of the reader first, reader second, and readers consensus.

Group (n=)	AUC (95%CI)			
	AI	AI+ Reader First-1	AI+Reader Second-1	AI+ Consensus
Overall	0.792 (0.660-0.925)	0.880 (0.793-0.968)	0.893 (0.809-0.976)	0.908 (0.832-0.984)
By age women, Y				
Younger (<55)	0.762 (0.588-0.936)	0.842 (0.719-0.964)	0.851 (0.730-0.971)	0.877 (0.770-0.995)
Older (≥55)	0.870 (0.683-1.000)	0.940 (0.814-1.000)	0.980 (0.919-1.000)	0.990 (0.951-1.000)

The results of the comparisons with radiologists’ assessments are presented in **Table 5**. AI algorithms alone achieved a specificity of 61.1% and a sensitivity of 80.6%. The specificity for Reader First-1 was 55.5%, and the sensitivity was 86.1%. The results of the combined assessment of AI and Reader First-1 showed a specificity of 72.7% and sensitivity of 91.7%. The performance showed significant improvements compared with AI alone (p<0.001) as well as the reader first-1 alone (p=0.006). The sensitivity of the combined assessment of AI and Reader First-1 was slightly higher than that of Reader Second-1 (91.7% vs. 88.9%). The diagnostic accuracy for the combination of AI algorithms and Reader First-1 was 85.2%, and for Reader Second-1, it was 85.2%. The performance of the combined assessment of AI algorithms and Reader Second-1 was better than the combination AI algorithms and Reader First-1, as was the combination of AI algorithms and reader consensus.

**Table 5:** Screening performance benchmarks for artificial intelligence algorithms and for radiologists among the 36 patients who received a diagnosis of malignant AD and 18 women who received a diagnosis of benign AD.

Benchmark	Reader first-1	Reader second-1	AI	AI+ Reader first-1	AI+Reader Second -1	AI+Consensus
Specificity	55.5%	77.8%	61.1%	72.2%	88.9%	88.9%
Sensitivity	86.1%	88.9%	80.6%	91.7%	88.9%	83.3%
Accuracy%	75.9%	85.2%	74.1%	85.2%	88.9%	85.2%
PPV	79.5%	88.9%	80.6%	86.8%	94.1%	93.8%
NPV	66.7%	77.8%	61.1%	81.3%	80.0%	72.7%

PPV, positive predictive value; NPV, negative predictive value.

## Discussion

The current work demonstrates that AD remains a challenging task for readers, even in the digital era. Radiologists have been reported to demonstrate poor performance in differentiating between benign and malignant tissues (25, 26). The best performance of Reader First-1 had an overall AUC of 0.733 for the detection of cancer *via* diagnostic mammography. The two other first readers had overall AUCs of 0.652 and 0.655. The best performance of Reader Second-3 had an overall AUC of 0.884 for the detection of malignant AD *via* diagnostic mammography. The two other second readers had overall AUCs of 0.875 and 0.882. The AI performance showed an overall AUC of 0.792. The computer algorithm reached, and in some comparisons surpassed, the performance level of junior radiologists in assessing malignant AD on mammography. However, the performance levels for AI algorithms did not outperform the assessments of all senior breast radiologists or consensus.

There was additional improvement in performance when models and junior doctors had access to clinical variables, including the patients’ age and breast density. The subgroup analysis of AUCs in our study showed a decreased performance for younger vs. older women and for higher vs. lower breast density on mammography. This is in line with prior studies showing decreased mammographic sensitivity in younger women and those with higher mammographic density (27, 28). Dense glands in Asian women may increase the difficulty of detecting AD. However, for senior doctors who have rich experience in breast imaging diagnosis, the influence of patient age and gland density on diagnosis can be ignored. In addition, the patient’s clinical data, clinical history, and prior imaging examinations were not adequately referenced. The machine learning and deep learning (ML-DL) models that combined information from both images and clinical data performed better than the ML-DL models trained on images or clinical data alone (29). The AI algorithms did not exploit the use of prior imaging examinations from the same women. The findings suggest that for future algorithm development, prior images from the same women should be used to detect early breast cancer. The changes can be observed by comparing prior and subsequent imaging examinations, especially for AD detection.

A computer algorithm that performs at or above the level of a radiologist in mammography screening assessments could improve the effectiveness of breast cancer screening (27). Detection algorithms for mammography that use the expertise of a reader and AI can identify more positive cases than two readers combined (28, 30). We know that combining assessments can improve the performance based on double-reading diagnostic programs. When assessing the combination of junior reader and AI algorithms, we achieved a markedly higher performance than a junior reader and an AI algorithm alone, increasing the overall AUC value from 0.733 to 0.880. When an AI algorithm is used by a junior reader, we obtained higher specificity and lower false positives; more true positive cases would likely be found. However, a much larger proportion of false-positive results still existed even when junior readers used AI algorithms. We found that the use of an AI algorithm by a senior reader did not achieve markedly higher performance than a senior reader alone. Likewise, when combining the algorithm with the consensus, we found no clear advantage over a senior reader alone.

The results from our study underscore the potential of using deep learning methods to enhance the overall accuracy of pretest mammography for malignant AD. There is a large gap in the diagnostic ability of radiologists in basic-level hospitals across the different regions of China. Radiologists are required to report on X-ray, CT and MR examination results, and even imaging technicians are required for this work some of the time. Our results suggest that adding AI to clinical mammography interpretation in settings with junior radiologists could yield significant performance improvements, with the potential to reduce health care system expenditures, address the recurring shortage of experienced radiologists, and reduce missed detection of early breast cancer.

## Limitations

This study has some limitations. We recognize that this combination of radiologist interpretation and AI algorithms is currently only theoretical in nature. We did not study the interaction of a human interpreter with AI algorithm results or how AI could influence radiologists’ final assessments areas that require greater research efforts. Furthermore, additional time was required for the radiologist to consider each CAD-marked area.

## Data Availability Statement

The raw data supporting the conclusions of this article will be made available by the authors, without undue reservation.

## Ethics Statement

The studies involving human participants were reviewed and approved by Ethics committee The Second Clinical College of Guangzhou University of Traditional Chinese Medicine. The ethics committee waived the requirement of written informed consent for participation.

## Author Contributions

BL and DC had full access to all of the data in the study and take responsibility for the integrity of the data and the accuracy of the data analysis. YWis the first author. Concept and design: YW, YT, YL, GY,DC, BL Acquisition, analysis, or interpretation of data: YW, YT, YL, YH, GY, DC, BL Drafting of the manuscript: YW, BL Critical revision of the manuscript for important intellectual content: DC, BL Statistical analysis: YW, Obtained funding: The authors states that this work has not received any funding. Administrative, technical, or material support: DC, BL Supervision: DC, BL. All authors contributed to the article and approved the submitted version.

## Conflict of Interest

Author YT was employed by Boston Meditech Group and Shanghai Yanghe Huajian Artificial Intelligence Technology Co., Ltd and DC was employed by Boston Meditech Group.

The remaining authors declare that the research was conducted in the absence of any commercial or financial relationships that could be construed as a potential conflict of interest.

## Publisher’s Note

All claims expressed in this article are solely those of the authors and do not necessarily represent those of their affiliated organizations, or those of the publisher, the editors and the reviewers. Any product that may be evaluated in this article, or claim that may be made by its manufacturer, is not guaranteed or endorsed by the publisher.
